# NumtS colonization in mammalian genomes

**DOI:** 10.1038/s41598-017-16750-2

**Published:** 2017-11-27

**Authors:** F. M. Calabrese, D. L. Balacco, R. Preste, M. A. Diroma, R. Forino, M. Ventura, M. Attimonelli

**Affiliations:** 1 Department of Biology, University of Bari, Bari, 70124 Italy; 2 School of Pharmacy, University of Nottingham, University Park, Nottingham, NG7 2RD UK; 3 Department of Biosciences, Biotechnologies and Biopharmaceutics, University of Bari, Bari, 70124 Italy

## Abstract

The colonization of the nuclear genome by mitochondrial DNA is an ongoing process in eukaryotes and plays an important role in genomic variability. Notwithstanding the DNA sequence availability of about 100 complete eukaryotic genomes, up to now NumtS distribution has been fully reported for a small number of sequenced eukaryotic species. With the aim to clarify the time and way of NumtS evolution, we explored the genomic distribution of NumtS in 23 eukaryotic species using an intra/interspecies *in silico* approach based on a cross-species similarity search and deeply investigate the evolution of NumtS in mammals. The intra- and interspecies analysis underlined how some mitochondrial regions that populated nuclear genomes can be considered as hotspots. Considering the large amount of NumtS we found in platypus and opossum genomes, we hypothesized the occurrence of an earlier colonization that happened prior to the Prototherian/Therian mammal divergence, approximately 160–210 million years ago. These events are still detectable due to the species-specific dynamics that have affected these genomes. Phylogenetic analyses of NumtS derived from two different mitochondrial DNA loci allowed us to recognize the unusual NumtS evolution that acted differently on primate and non-primate species’ genomes.

## Introduction

Mitochondria are remnants of α-proteobacterial endosymbiosis, and following this process, the mitochondrial genome (mtDNA) underwent over time an important size reduction caused by horizontal gene transfer to the nucleus^1^. This transfer is an important mechanism for eukaryotes and their genome evolution^2–4^; indeed, fragments in the nuclear genomes of mtDNA have been recognized and termed as NumtS (Nuclear mitochondrial Sequences)^5–7^. Studies on *Saccharomyces cerevisiae* suggest that the intracellular escape of NumtS from mitochondria and their integration within the nuclear genome occurred at a frequency of 10^−3^ to 10^−4^ per cell per generation^8,9^. A widely accepted hypothesis proposed that NumtS insertion could occur in double-strand breaks by non-homologous end joining (NHEJ) machinery with or without the requirement of short microhomology^10,11^. Some NumtS integration events in human and chimpanzee were shown to be associated with microhomology and short insertions and deletions (indels), typically observed in the NHEJ pathway of the DNA double-strand break-repair mechanism^12^. NumtS can be lost or the insertion events may result from the *de novo* arrival of DNA from mitochondrion or depend on duplications involving already fixed fragments, thus generating more complex rearrangements in the whole nuclear genome^13–16^. Considering the whole nuclear genome variability, population bottlenecks strongly contributed to shaping the architecture of the human nuclear genome and it has been proposed that different NumtS colonizations may have been concomitant and dependent events^17^.

According to this hypothesis, random genetic drift may be considered as the driving force for NumtS acquisition. Under neutral selective pressures, this force becomes pronounced during profound reductions in population size and would have an influence on the acquisition of DNA changes.

NumtS are highly polymorphic in terms of sequence, homo/heterozygosis status, and presence/absence at a specific locus^18–20^. Thanks to their intraspecies variability, NumtS may be considered population markers^17,20,21^ as confirmed in human by PCR validation and sequencing^22–25^. Improvements in sequencing approaches enhanced better genome refinement and at the same time offered the possibility to rely on a more accurate NumtS annotation. The NumtS density was already explored in other species like human, mouse, macaque^25,26^, beetle^27^ and honeybee^28^ and was found exceptionally high in honeybee. To shed light on the generation and evolution of NumtS in mammals, we created and compared at the sequence level the full NumtS collection (the NumtSome) for 23 eukaryotic species, whose genomic sequences were annotated at the UCSC Genome Browser (http://genome.ucsc.edu/). Our analyses highlighted species-specific signatures in NumtS colonization and defined hotspot regions in mitochondrial DNA (mtDNA) as being more prone to create NumtS. In particular, we detected a high-coverage mtDNA region in the platypus genome, which we considered as a starting point (seeding) for Numts colonization process. Moreover, studying the phylogenetic relationship among NumtS, we were able to depict NumtS evolution patterns that differently affected primate and non-primate species.

## Results

### The species batch

**Figure 1:**
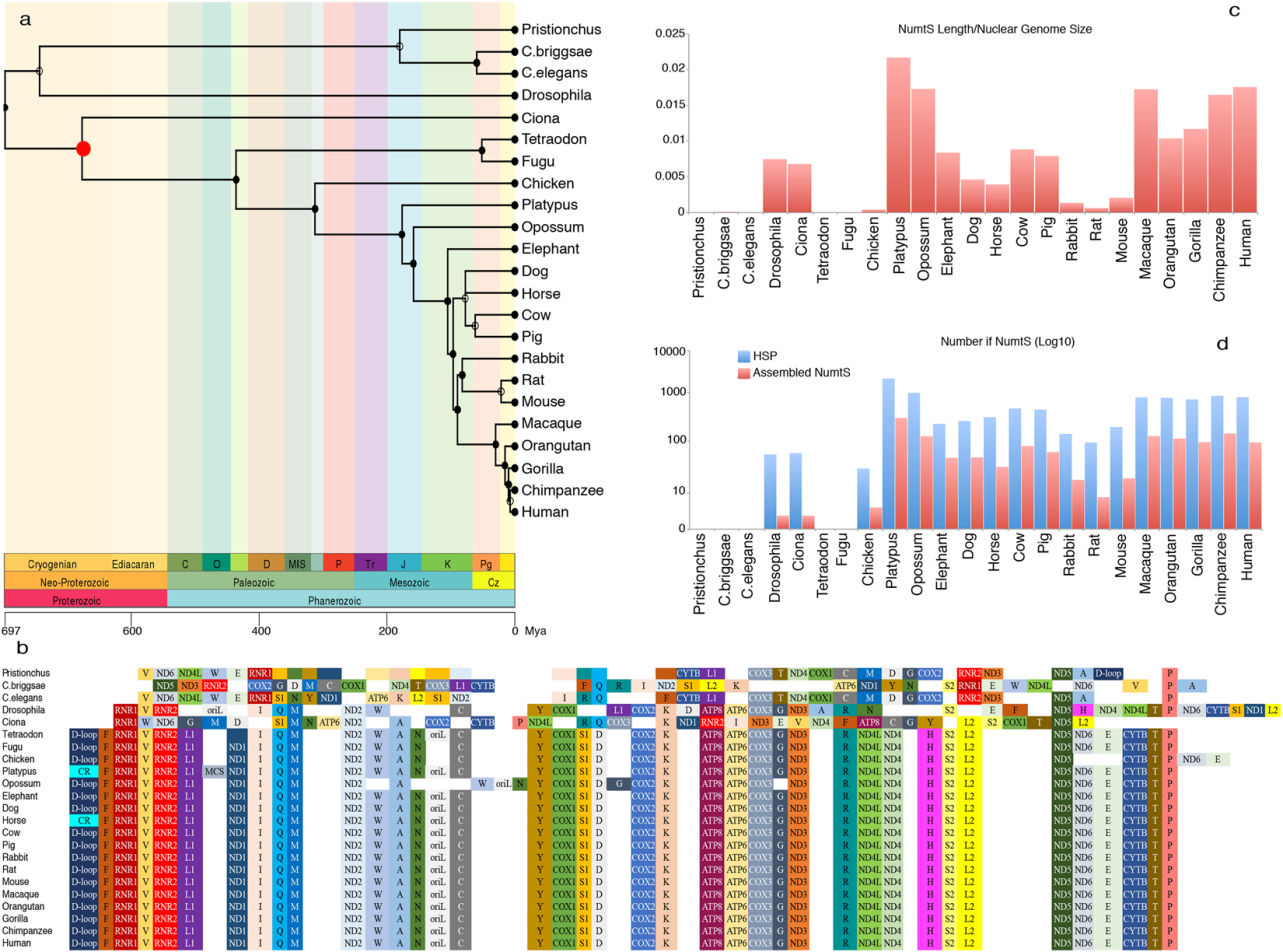
Schematic representations of species divergence and NumtS content in the analysed species batch. (**a**) Phylogenetic divergence among the considered species. Geological timescale and time (Mya) are also reported. The cladogram was obtained using the TimeTree database^40^. The red circle indicates the divergence node between the Chordata and the Nematoda-Arthropoda phyla, the latter exhibiting a different mitochondrial loci organization (part b). (**b**) The mitochondrial loci order qualitatively compared among the 23 species. White colored blocks were inserted when the placement of the mt loci order did not match the conserved loci position. Loci data were retrieved from the feature tables of the GenBank-relative mitochondrial entries (Accession numbers reported in Table 1). (**c**) Number of NumtS found in each species, normalized to the nuclear genome size. Blue bars represent the number of single HSPs, while red bars refer to assembled NumtS. (**d**) NumtS percentage on the nuclear genome total length.

The colonization of the nuclear genome by mtDNA is an ongoing process that is difficult to elucidate. With the aim of exploring this evolutionary conundrum, NumtSomes were characterized in 23 species selected using two criteria: the availability of complete genomic sequences reported in the UCSC Genome Browser and their phylogenetic proximity (Table 1, Fig. 1a). The organization of mtDNA loci showed great differences between Nematoda-Arthropoda vs. Chordata phyla (Fig. 1b).

**Table 1:** Features of the 23 species analysed in this study.

Species	Common name	Assembly	Nuclear genome size (Mb)	Mitochondrial genome Accession #	chrM size (bp)	NumtS HSP number
Pristionchus pacificus	Pristionchus	priPac1	133.64	NC_015245.1	15,955	52
Caenorhabditis briggsae	C.briggsae	cb3	108.48	NC_009885.1	14,420	59
Caenorhabditis elegans	C.elegans	ce6	100.28	NC_001328.1	13,794	1
Drosophila melanogaster	Drosophila	dm3	139.49	U37541.1	19,517	43
Ciona intestinalis	Ciona	ci2	172.99	NC_004447.2	14,790	46
Tetraodon nigroviridis	Tetraodon	tetNig2	342.4	DQ019313.1	16,448	5
Takifugu rubripes	Fugu	fr2	393.31	NC_004299.1	16,447	7
Gallus gallus	Chicken	galGal3	1,098.77	NC_001323.1	16,775	21
Ornithorhynchus anatinus	Platypus	ornAna1	1,995.61	NC_000891.1	17,019	4412
Monodelphis domestica	Opossum	monDom5	3,598.44	NC_006299.1	17,079	939
Loxodonta africana	Elephant	loxAfr3	3,196.74	NC_000934	16,866	198
Canis lupus familiaris	Dog	canFam2	2,528.45	NC_002008.4	16,727	302
Equus caballus	Horse	equCab2	2,474.93	NC_001640.1	16,660	278
Bos taurus	Cow	bosTau6	2,670.42	NC_006853.1	16,338	432
Sus scrofa	Pig	susScr2	2,262.48	NC_000845.1	16,711	403
Oryctolagus cuniculus	Rabbit	oryCun2	2,737.45	NC_001913.1	17,245	239
Rattus norvegicus	Rat	rn4	2,826.22	NC_001665.2	16,313	81
Mus musculus	Mouse	mm9	2,745.14	NC_005089.1	16,299	169
Macaca mulatta	Macaque	rheMac2	3,097.57	NC_005943.1	16,564	745
Pongo pygmaeus abelii	Orangutan	ponAbe2	3,441.23	NC_001646.1	16,499	902
Gorilla gorilla gorilla	Gorilla	gorGor3	3,063.66	NC_001645.1	16,364	674
Pan troglodytes	Chimpanzee	panTro3	3,307.94	NC_001643.1	16,561	914
Homo sapiens	Human	hg19	3,137.14	J01415.2	16,569	764

Genome details of selected species plus intraspecies High Scoring Pair (HSP) calls in the last column.

### Genome quality and purging

In order to remove possible artefacts made up only by mtDNA, the Blast outputs for the 23 species were subjected to a purging process. This led to the removal of NumtS located in contigs whose mtDNA content spanned over 80% of their total length (occupancy percentage) (Supplementary Table S1).

In platypus 103 contigs (2.47% of the whole genome) were discarded as they were found to be entirely composed of mtDNA (Supplementary Table S2). Notwithstanding the high percentage of discarded NumtS (50.83%), platypus still was the species with the greatest number of NumtS (Fig. 1c,d and Supplementary Figure S1).

In order to test whether NumtS content correlated with contig- or scaffold-defining assembly quality values, we computed the Spearman’s correlation coefficient matrix and we calculated for each ranking dependence a statistical corrected significance p-value (Supplementary Figure S2A). By analysing the whole set of species, we could not detect any correlation between NumtS quantity and the genomic statistical parameters such as contig/scaffold-N50/L50 or genomic gap size. As the species set used for the correlation metrics changed, no significant correlations were found between NumtS number and genomic statistical parameters (Supplementary Figure S2B-D).

### Intra- and interspecies NumtS compilations

By running all crossed Blasts between nuclear and mitochondrial genomes of the 23 selected species, a total number of 23 intra- and 506 interspecies NumtS compilations were generated (Table 1 and Supplementary Table S3). A higher number of NumtS with respect to all other species was observed in primate genomes: macaque, orangutan, gorilla, chimpanzee, and human. Two exceptions to this evidence were found in the platypus and opossum genomes with the first species showing a higher number of NumtS than all the other species (Fig. 1c,d). The highest interspecies NumtS mean lengths were found in elephant, followed by the three primates human, chimpanzee, and macaque (Supplementary Table S4A). The mean similarity of interspecies NumtS High Scoring Pairs (HSPs) approximately ranged between 72% and 87% (Supplementary Table S4B).

### NumtS and repetitive elements

To investigate the co-occurrence of repetitive elements (REs) and NumtS, we analysed the repeat content in NumtS loci and both flanking regions (2 kbp long). After normalizing the RE number on the total NumtS amount, flanking regions were shown to exhibit a higher RE number with respect to NumtS loci; moreover, primate genomes did exhibit a smoother increase in REs encompassing NumtS loci and flanking regions (Supplementary Figure S3, Supplementary Table S5). The REs are almost equally distributed in both flanking regions in all species with the exception of platypus and opossum genomes, where the 5′ flanking regions were enriched in REs (Supplementary Table S5). Positive correlation (p-value = 6.25E-03) was estimated between NumtS and RE number in both flanking regions (Supplementary Figure S4).

### Nuclear genome coverages

**Figure 2:**
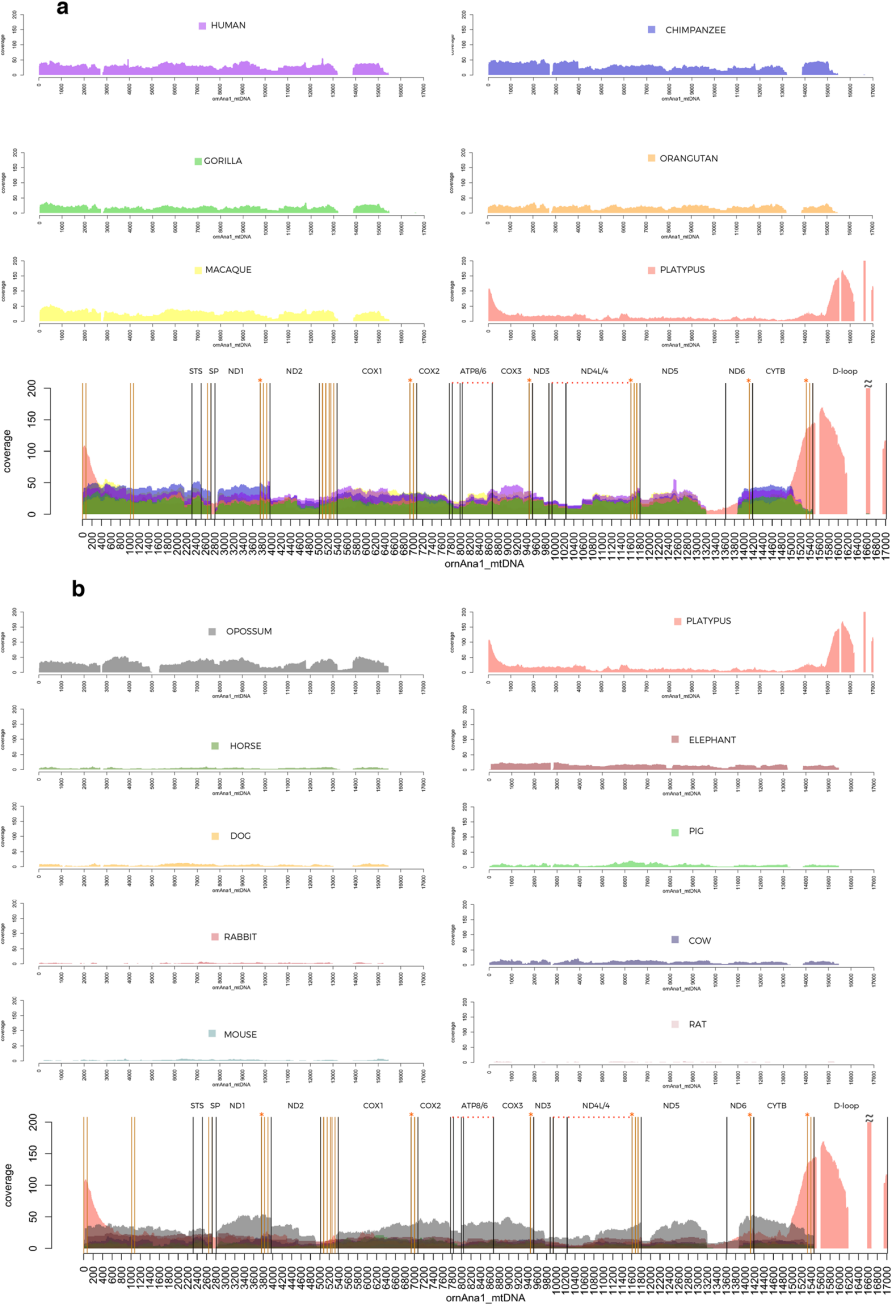
Platypus mitochondrial genome cross-coverage graphs. (**a**) Platypus mitochondrial cross coverage on primate and platypus whole nuclear genomes. (**b**) Platypus mitochondrial cross coverage on platypus and other mammalian (no primates) whole nuclear genomes. Mitochondrial cross NumtS coordinates were used to plot coverage profiles and area graphs have been layered in transparency. Mitochondrial loci and tRNA genes start and end points were drawn as vertical lines (in black and orange, respectively). The orange stars indicate the DNA bases where two mt loci overlap.

In order to evaluate mitochondrial loci contribution to the genome colonization, we performed a coverage analysis based on intra- and interspecies NumtS detection. Fixing one mitochondrial genome at a time, each intra- and interspecies set of crossed Blast outputs was plotted reporting for each position of the mtDNA the per-base counting occurrence on the nuclear genome. On average, the nuclear coverage obtained for primate genomes was higher than those observed with the other species (Fig. 2a) with the exception of platypus and opossum nuclear genomes, which were the highest (Fig. 2b).

The interspecies NumtS detection that allowed for the recognition of specific mitochondrial regions in the nuclear genome, highlighted an increase of coverage in the platypus nuclear genome with whatever mtDNA it was blasted against; subsequently, the opossum nuclear genome showed more continuously spanned coverage and most of mitochondrial loci with high coverage were in common with the platypus nuclear genome (Supplementary Figure S5).

Except for some negligible cases, the interspecies analysis did not sum up big discrepancies in NumtS number with respect to the intraspecies analysis (Supplementary Table S3).

The intraspecies coverage analysis in platypus identified lower peaks in different mitochondrial loci (Fig. 2a); one of these corresponding to the ND2 locus was also confirmed in the mammalian coverage (Fig. 2 b). Of note, some mitochondrial loci exhibited an exclusive coverage on the platypus nuclear genome. This was the case of the mitochondrial region spanning ND5/ND6 loci, the control region, and the specific Monotremata spacer “SP” exclusive of platypus and echidna mitochondrial genomes. In particular, the platypus control region (16586–16675) showed the highest per-base occurrence (>700). Moreover, a portion of platypus sequence (from 33 bp to 699 bp inclusive of the tRNA-phe and the rRNA 12S) shared high similarity (74%) with the echidna mtDNA, while the remaining part did not match any entry in the entire NCBI Blast database (data not shown). The interspecies mtDNA chimpanzee coverage analysis revealed that ATP8 and one portion of ND1 were not detected in any of the mammalian nuclear genomes considered here.

### The NumtS compilations as UCSC custom tracks

In order to provide a framework for the exploitation of intraspecies data, the species compilations were implemented as UCSC Genome Browser annotation tracks within the “Variation and Repeats” group, as was previously done for the tracks relative to human (hg18 and hg19 assemblies)^25^, other primates (i.e., chimpanzee (panTro3 release) and macaque (rheMac2 release)), and mouse (mm9 release)^26^. For each analysed species four different tracks were produced: i) “NumtS” reporting nuclear mapping position of purged HSPs; ii) “Assembled NumtS” reporting merged NumtS if located on the same strand and no more than 2 kbp apart; iii) “NumtS on mitochondrion” reporting mitochondrial mapping position; and iv) “NumtS on mitochondrion with mismatches” showing the single-nucleotide polymorphism pattern, which differentiates the nuclear and the mitochondrial counterpart^25,26^ (Supplementary Table S6).

### Platypus NumtS dating

Due to the distribution of NumtS in the platypus genome, we attempted to date some of their insertion events. We used the Hmmer software^29^ to infer if a compositional bias among NumtS in platypus and their flanking regions could be evidence of how much these fragment compositions homogenised within the nuclear context (Supplementary Figure S6, Supplementary Table S7). Hmmer analysis of platypus intraspecies NumtS contributed to estimate a relative ancestry among them. A narrow subset of 43 NumtS and their flanking regions was selected (Methods). Each NumtS was aligned with a profile derived from the platypus mitochondrial genome and then against a profile built for its own flanking regions.

The coefficient showing the discrimination between fragments that are more similar to mitochondrial sequences and those more similar to the nuclear sequences was calculated as the difference between the score on the mitochondrion profile and the score on flanking region profiles. The Hidden Markov Models profiles (HMM) mitochondrial and flanking region score differences are meaningful for relatively new insertion events, if positive, or old, if negative (Supplementary Table S7). Three of these were more similar to the mitochondrial genome, thus more recently inserted, whereas the remaining three showed a negative score and hence a more ancestral insertion event.

### NumtS phylogeny

**Figure 3:**
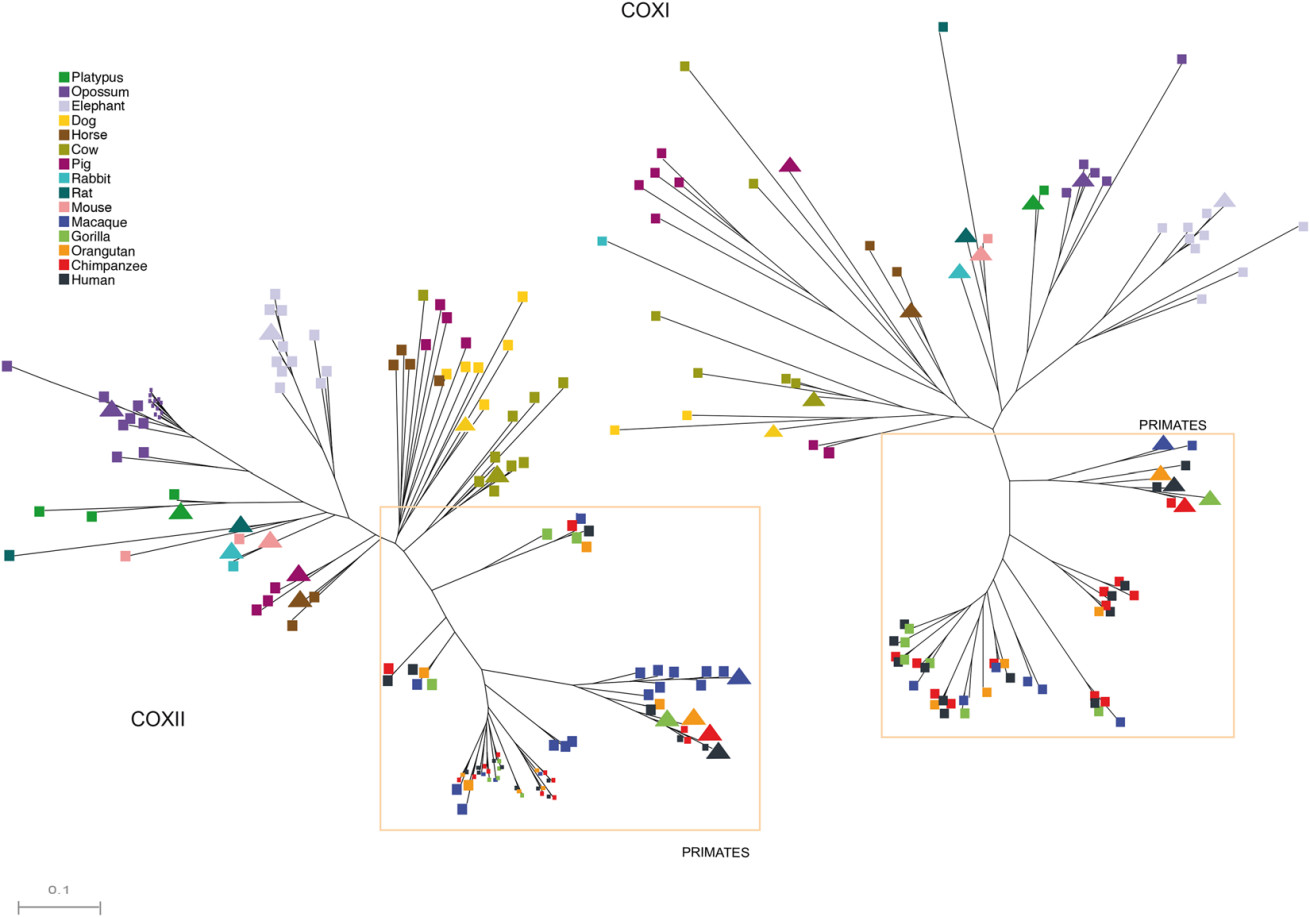
Mammalian NumtS phylogeny relative to the COXI and COXII mitochondrial loci. Mitochondrial sequences in each species are identified by triangles. Due to the abundance of NumtS analysed and in order to appreciate closely related branches, some random squares relative to primates and *M*.*domestica* branches are shown smaller than the others.

In order to study the evolution of NumtS, we conducted a phylogenetic analysis using regions of NumtS mapping onto COXI and COXII loci (Fig. 3). The multi-alignments were used for the approximately maximum-likelihood phylogenetic tree construction. Both the obtained trees clearly highlighted two different clusters in non-primate mammals and in primate species. While non-primate mammals showed species-specific clusterization, primate NumtS was intermingled among them with the exception of macaque, which showed both patterns. Of note, the mitochondrial COXI- and COXII-derived NumtS in primates showed a similar clusterization.

**Figure 4:**
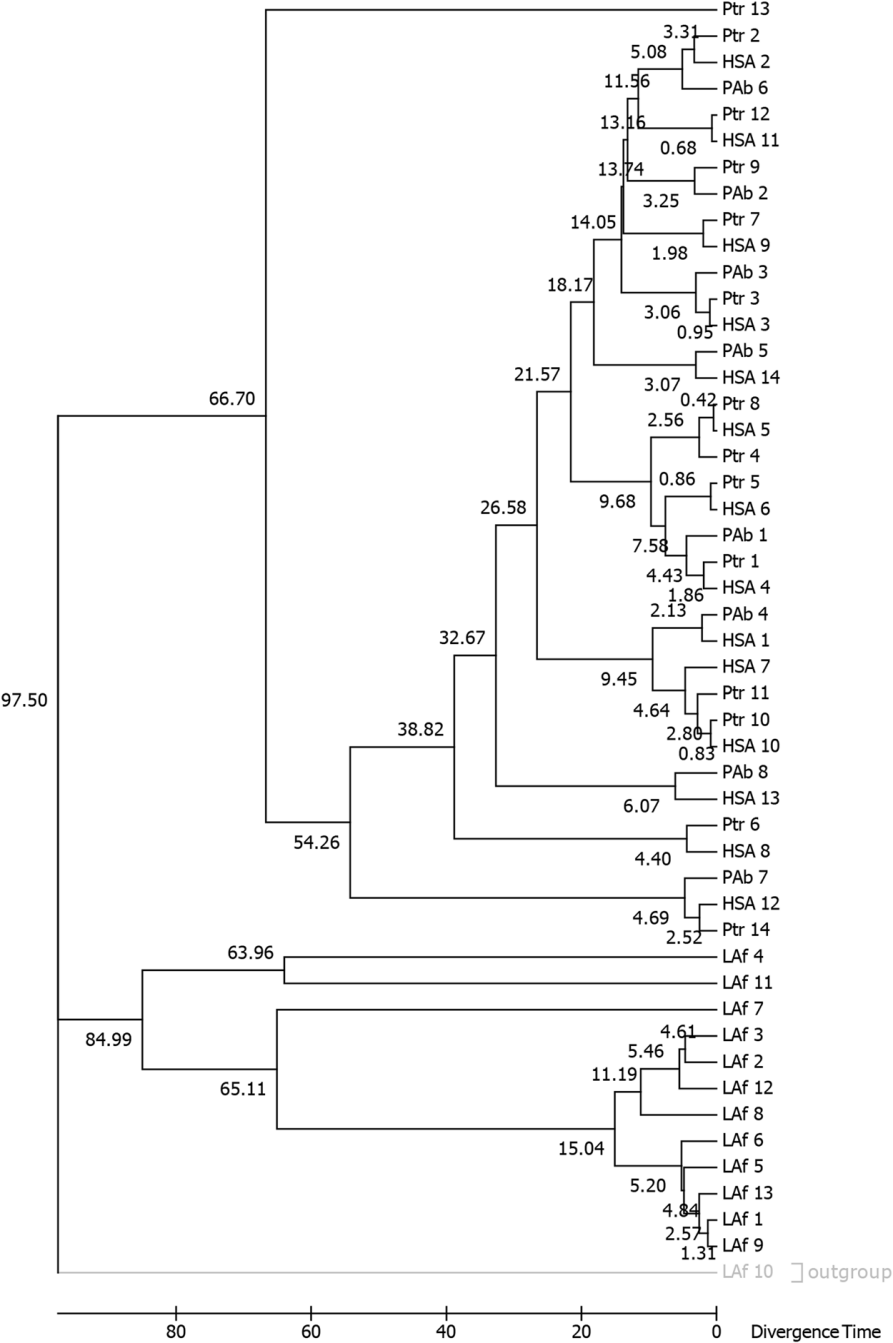
NumtS timetree. The same sets of primate and *Loxodonta africana* (elephant) NumtS spanning the COX1 mitochondrial locus (Fig. 3) were used to calculate the divergence among primate nodes. The divergence of elephant NumtS was fixed in a 95–105 Mya time window.

With the aim to date NumtS insertion events in primates, we aligned human, chimpanzee, orangutan and elephant COXII-derived NumtS, which roughly span from 7000 to 7700 on their mitochondrial genomes (Fig. 4). Fixing at 100 million years ago (Mya), the elephant divergence time, we were able to date the evolution of the different events in primates and elephant. Importantly, in line with the COXI-II analysis, elephant NumtS clustered separately from primate NumtS.

## Discussion

The analyses we present here are aimed at understanding NumtS colonization events by both intra- and interspecies mapping and sequence comparisons in mammals. A massive percentage of NumtS was detected in platypus and opossum after the filtering step that removed about 50% of HSPs in platypus. Noteworthy, 2.47% of contigs in platypus genome were pure mtDNA thus possibly suggesting a massive enrichment of mtDNA in the sequenced genomic DNA. Moreover, intraspecies platypus NumtS analysis revealed a high occurrence (>700) of a small portion (100 bp) of the mtDNA control region. It is plausible that this untranslated portion represented a hot-spot region in the mtDNA for NumtS genesis and was used as a seeding unit during colonization within the platypus nuclear genome. Since we revealed such differences in NumtS coverage for this region only in platypus, we speculated that this dynamic was platypus-specific.

Noteworthy, purging criteria recently published highlighted the possibility to reduce the raw number of NumtS and nupts in genomic compilations^30^. In this regard, in our protocol we defined as criteria the fragment spanning length, orientation and presence of RE between fragments (two HSPs interrupted by a RE were called as single Numts), thus allowing to reduce the overestimation effect on the NumtS calls.

In order to obtain comprehensive compilations of NumtS, we ran an interspecies analysis among the selected species. Regardless of the mtDNA used, platypus showed high coverage of STS/ND1, ND6/CYTB, and ND5 mitochondrial loci. A similar but smoother pattern was observed in opossum genome analyses. We hypothesized that the presence of these high-coverage interspecies regions is the result of an earlier colonization on these nuclear genomes, which occurred before the Prototherian/Therian mammalian divergence followed by a mutational “frozen” step. This could have preserved the integrity of NumtS regions, thus allowing the detection of events otherwise lost in other species. More importantly, this speculation may reflect the isolation of platypus as an evolutionary deadwood branch^31^.

Remarkably, other than platypus and opossum, both intra- and interspecies data showed sensibly higher coverage in primates than all of the other species. This supports the hypothesis proposed by Gherman *et al*., where most NumtS seemed to have been accumulated in a 10-million-year window centered on 54 Mya between the New World monkey and Old World monkey evolutionary transition^17^.

The inspection of NumtS loci and their flanking regions showed NumtS positive correlation with REs. We hypothesized that REs may have played a central role in NumtS fixation or, vice versa, that REs may have accumulated in regions enriched in NumtS, in line with segmental duplication evolution observed in primates^32^. Furthermore, since REs constitute a background noise in understanding NumtS compositional homogenization, we searched the platypus genome for NumtS devoid of REs in the flanking regions and detected a subgroup that poorly homogenized within genomic context, thus indicating more recent insertions.

In order to shed light on the evolution of NumtS and timescale of the colonization events, we performed a phylogenetic analysis on two mitochondrial loci, COXI and COXII, and found two different patterns of evolution: (i) intermingled NumtS clusters in primates and (ii) species-specific clusters relative to mammals other than primates in both the trees, particularly noticeable for platypus, opossum, and elephant NumtS. Of note, macaque showed both patterns in the COXII phylogenetic tree. Contextually, setting the elephant–great ape divergence between 90 and 105 Mya, we were able to detect and distinguish human-chimpanzee-orangutan and elephant-specific events. We speculated that in primates, before their branch species divergence, colonization by mtDNA occurred in the common ancestor while in species other than primates, gene conversion and/or species-specific duplication events could have acted to homogenize NumtS at the sequence level.

Overall, our data shed light on different aspects of NumtS genesis and evolution, such as the presence of hotspot regions in mitochondrial genomes and that platypus and opossum genomes maintained a “frozen” mutational status allowing the detection of the more ancient insertion of mitochondrial fragments. Moreover, we found pronounced differences between primate and non-primate mammalian NumtS evolution, thus indicating distinctive evolutionary mechanisms for the insertion time and colonization in these two groups. Further analyses are necessary to define and specifically characterize if the evolutionary forces could have acted any differently.

## Methods

### Creating NumtS compilations

A BlastN similarity search between nuclear and mitochondrial genomes was run on 23 selected species (Table 1) and was based on all possible intra- and interspecies blasts (crossed blasts). The similarity search between mtDNA and nuclear DNA in each run was performed with BlastN^33^ version 2.2.26 using the e-value “10^−3^” and the “−b” option (number of database sequences to show alignments for) set to 200,000 to allow for the detection of NumtS in unfinished genomes such as platypus (ornAna1), which contained a summed number of chromosomes, contigs, and ultracontigs close to this number. The entire set of HSPs obtained as BlastN output was filtered to prevent possible mitochondrial contamination removing (i) the contigs whose composition in mt bases was higher than 80% and (ii) hits on random and unknown chromosomes thus generating the NumtSome.

### Intraspecies NumtS tracks within UCSC Genome Browser

Following the protocol described by Simone *et al*. in 2011^25^, the intraspecies NumtSome produced by applying BlastN were used to generate four types of UCSC Genome Browser tracks. Intraspecies blast hits with the same orientation were joined together when their position did not map further than 2 kbp or, based on the circumstance, they were intermingled by a unique RE even if it spanned longer than 2 kbp^25,26^.

All NumtS tracks were generated in the Browser Extensible Data (BED) format, apart from the “NumtS on mitochondrion with mismatches” track. In this case, the alignments resulting from the LASTZ software^34^ were run with default parameters and converted to the Binary Sequence Alignment/Map (BAM) format.

Hyperlinks pointing to the UCSC Genome Browser custom annotation tracks were created to show our NumtS track files stored on the public server folder. In order to prevent conflicts with older temporary cache stored files, the supplied addresses need to be run in a new incognito or private window. Both the nuclear and the mitochondrial NumtS items are interchangeably connected through an external HTML link, which allows shifting the genomic context from mtDNA to its counterpart on the nuclear chromosome.

### Repeated elements and NumtS co-occurrence

RepeatMasker database elements were retrieved in each species from the “golden path” ftp download website (http://hgdownload.cse.ucsc.edu/goldenPath/). Coordinate co-occurrences were computed with BedTools suite version 2.26.0 (http://bedtools.readthedocs.io/en/latest/content/bedtools-suite.html).

### Intra/interspecies mtDNA coverage graphs

A custom python script (available upon request) was used to parse interspecies blast output that was then plotted in the R environment through the “polygon” function. Coverage graphs were produced by considering the mitochondrial genome as fixed and observing its per-site occurrence on different nuclear genomes at a time. Two coverage graphs were produced for the mtDNA of platypus—the first group was composed of primate genomes, while the second encompassed various mammals. The mitochondrial genome coverage of opossum, cow, dog, horse, pig and chimpanzee was produced by considering the same nuclear genomes that were considered in the platypus mtDNA coverage graph. Multiple layer polygons were produced at a defined density in order to make them almost transparent.

### Inference of NumtS insertion events time

The Hmmer (http://hmmer.org/) software was used in order to date NumtS in platypus. A relative fixation time was also calculated in the same manner for a NumtS subset. This approach relied on probabilistic models termed HMM profiles, which turn a multiple sequence alignment into a position-specific scoring system suitable for searching databases for remotely homologous sequences^29^. Input files were prepared starting from repeated element, segmental duplication, interrupted repeat, and simple repeat tracks data annotated in the UCSC Genome Browser and the platypus NumtS custom annotation tracks. The intraspecies platypus NumtS subset used for the analysis was generated excluding all those NumtS containing the above-mentioned elements in their own sequence and in 1 kbp long flanking regions. Repeated elements were removed using the Galaxy “Subtract” tool from fragments containing NumtS and their flanking regions, then modified for processing with the Hmmer software.

In order to infer the relative time of NumtS insertion events, two Hmmer analyses were compared. The first analysis was performed on NumtS versus the mitochondrion HMM profile, whereas the second analysis was carried out on NumtS versus the HMM profile of their flanking regions. Profiles of flanking regions were generated for each NumtS. In the case of NumtS whose flanking sequences presented a high number of gaps due to incomplete assembly, a consensus profile built on all the other NumtS flanking regions was used.

### NumtS - genome assembling statistic correlation

Statistical correlations and significance were imputed in the R environment. Spearman’s correlations were calculated together with p-values by using the “rcorr” function within the Hmisc package (https://cran.r-project.org/web/packages/Hmisc) and plotted using corrplot (https://CRAN.R-project.org/package=corrplot, https://CRAN.R-project.org/package=corrgram). N50 and L50 values used for the correlation plots were retrieved from the NCBI assembly information page of each analysed species.

The significance threshold was normalized using Bonferroni correction for multiple tests.

### NumtS phylogenetic analyses

The phylogenetic trees are based on NumtS mapping the COXI and COXII loci and which spanned as much as possible the entire locus length. The two loci were chosen on the basis of the higher coverage peaks observed in Fig. 2a.

Multiple sequence alignments were performed using the Clustal Omega software^35^. Multiple alignment editing was performed with the multiple alignment editing program Jalview^36^. Phylogenetic analyses were carried out using the Approximate Maximum Likelihood analysis method from the FastTree software^37^, suitable for the construction of large phylogenies, implementing the neighbor-joining method with heuristics. The Java-based Archaeopteryx software^38^ was used to obtain a good graphical rendering of the phylogenetic trees. The evolutionary history was inferred by using the maximum likelihood method based on the Kimura 2-paramether model in the MEGA7 software^39^.

## Electronic supplementary material

Supplementary information
