# Supplementary material for: NumtS colonization in mammalian genomes
Source: Sci Rep. 2017 Nov 27;7:16357. doi: 10.1038/s41598-017-16750-2 (PMC5703718; doi:10.1038/s41598-017-16750-2)
Supplement: Supplementary file 1 — Supplementary information [file 41598_2017_16750_MOESM1_ESM.pdf]

Supplementary information

NumtS colonization in mammalian genomes.

Calabrese FM[1], Balacco DL[2], Preste R[3], Diroma MA[3], Forino R[3], Ventura M[a,1], Attimonelli M[b,3]

command-line for BLAST search:
blastall -p blastn -G -5 -E -2 -r 2 -q -3 -e 1e-03 -m 9 –I mt.fasta -d hg19.fa -o output_file

Contents:

Supplementary Figure S1. Testing of different thresholds in the purging step on platypus set of HSPs.

Supplementary Figure S2. Significance levels for Spearman's correlations between NumtS and genome statistical values.

Supplementary Figure S3. Repetitive elements count in NumtS loci and flanking regions.

Supplementary Figure S4. Significance levels for Spearman's correlations between NumtS and repetitive element number.

Supplementary Figure S5. Non-primate mammalian mitochondrial genome cross-coverage graphs.

Supplementary Figure S6. Hmmer analysis workflow.

Supplementary Table S1. Purging statistics.

Supplementary Table S2. NumtS mapping positions in platypus contigs.

Supplementary Table S3. Matrix of the number of BlastN high scoring pairs number.

Supplementary Table S4. BlastN high scoring pairs length and similarity matrix after purging.

Supplementary Table S5. Count of repetitive elements found in repeatmasker database.

Supplementary Table S6. UCSC tracks links.

Supplementary Table S7. Hmmer NumtS dating.

Supplementary Figure S1. The used 80% threshold was arbitrarily fixed in order to avoid contigs almost completely represented by mitochondrial bases and hence potentially assembly artefacts. In particular, decreasing of the threshold does not imply a sensible reduction of NumtS calls.

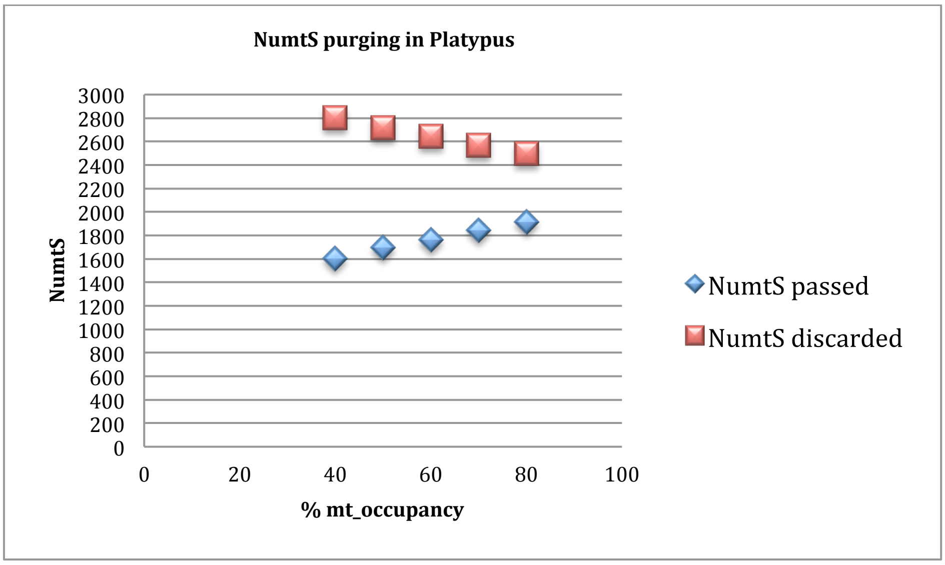

Supplementary Figure S2. Significance levels for Spearman's correlations between NumtS - NumtS before purging (NumtS_BP) and genome statistic values: scaffold N50, contig N50, total genomic gap length gap.gsize and number of contigs (contig_number). Statistical measure of the linear relationship was plotted in blue to red colour for positive to negative correlation, respectively. The areas of circles show the absolute value of corresponding Spearman's correlation coefficients. Numbers are indicative of the p-values and white flagged circles correspond to significant correlations. The significance threshold was calculated by using the Bonferroni correction for multiple testes. A) Correlation was calculated among all the 23 analysed species. B) Correlation was calculated excluding the human and mouse genomes. C) Correlation was calculated using all species but not primates. D) Correlation was calculated using only primates.

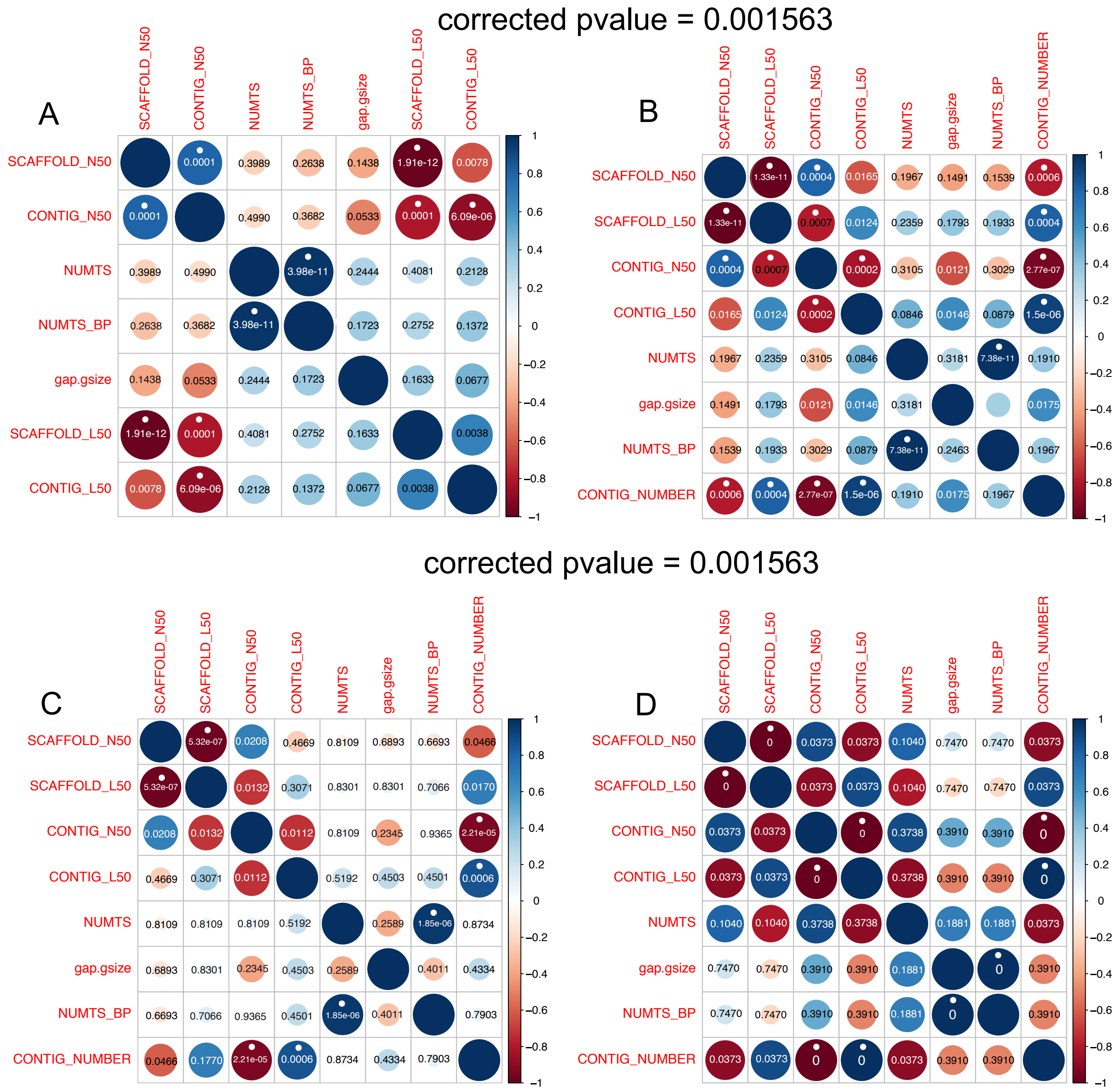

Supplementary Figure S3. Normalised repetitive elements count in NumtS loci and flanking regions. For each of the 23 species, a count of repetitive elements (normalised by NumtS count) is shown, for either NumtS loci and 5' and 3' flanking regions of 2 kbp each.

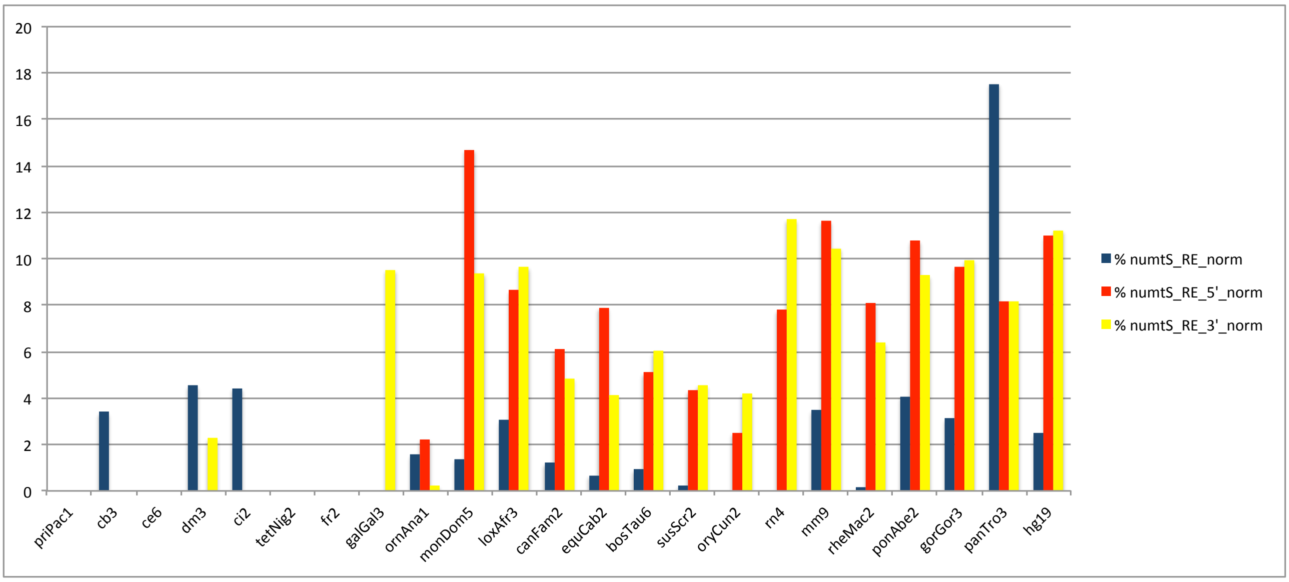

Supplementary Figure S4. Spearman's correlations between NumtS and repetitive element number. Correlation of NumtS elements with repetitive elements located in NumtS loci (RE_NumtS) and in 5' (RE_5) and 3' (RE_3) flanking regions. Statistical measure of the linear relationship was plotted in blue to red for positive to negative correlation, respectively. The areas of circles show the absolute value of corresponding correlation coefficients. Numbers are indicative of p-value; white flagged circles correspond to significant correlations. The significance threshold was calculated by using the Bonferroni correction for multiple testes.

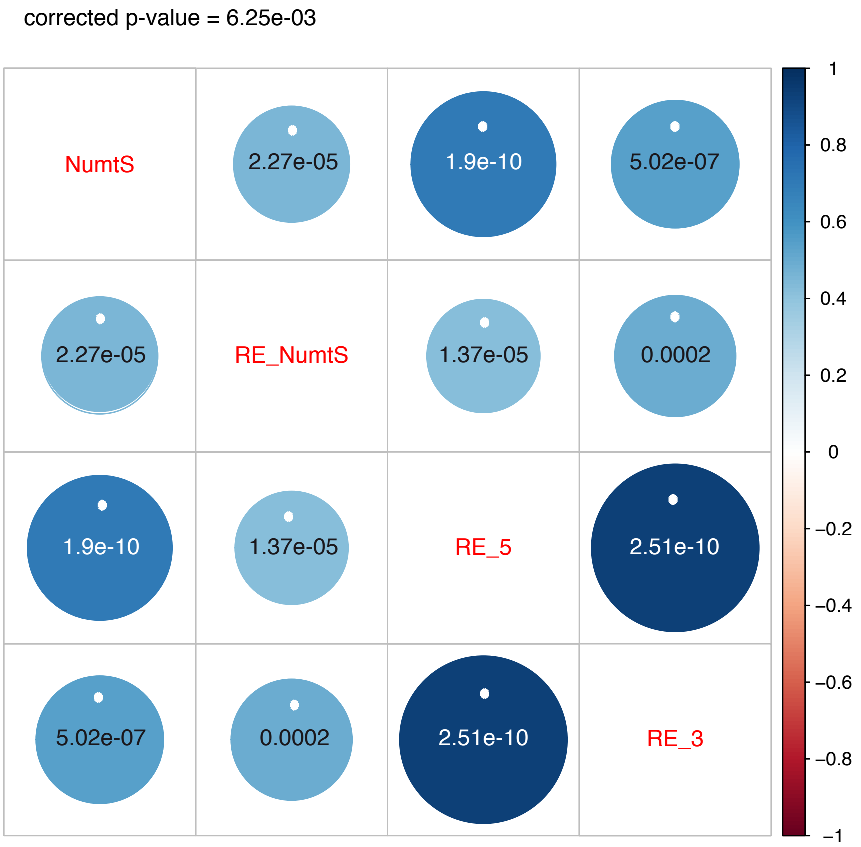

Supplementary Figure S5. Non-primate mammalian mitochondrial genome cross-coverage graphs. Opossum, cow, dog, pig, horse and chimpanzee mitochondrial genome cross coverage on platypus, opossum and mammalian whole nuclear genomes. Mitochondrial cross NumtS coordinates were used to plot coverage profiles and area graphs have been layered in transparency. The start and end points of mitochondrial loci and tRNA genes were drawn as vertical lines (in black and orange, respectively). The orange stars indicate the DNA bases where two mt loci overlapped.

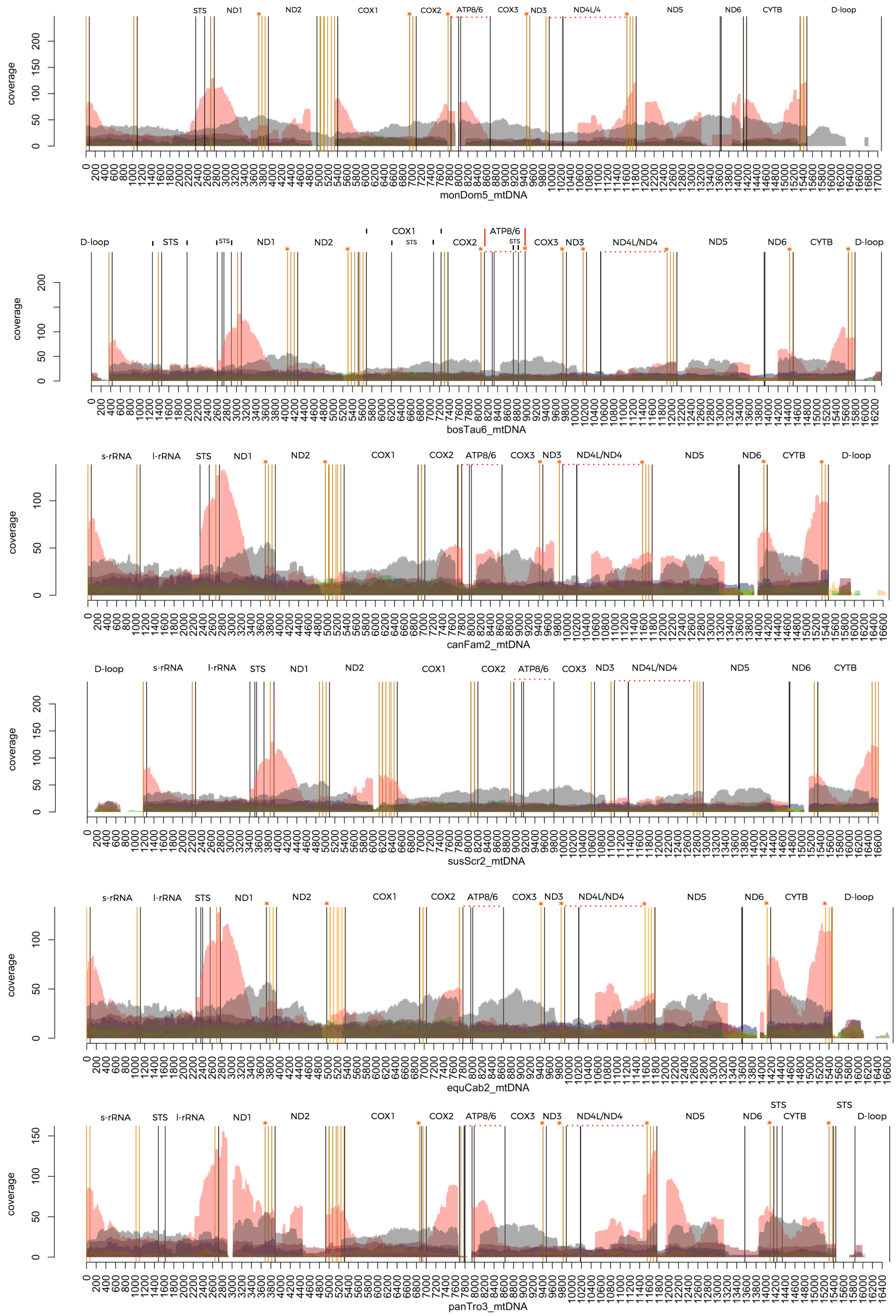

Supplementary Figure S6. Hmmer workflow is used to estimate NumtS relative insertion time.

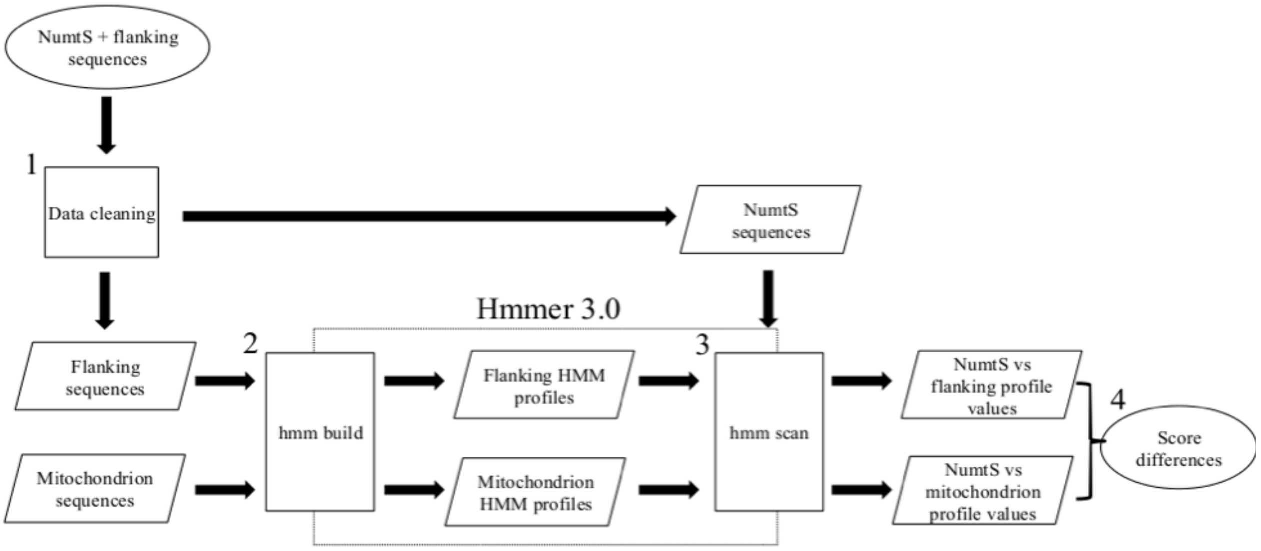

Supplementary Table S1. Statistics of High Scoring Pairs (HSPs) maintained after the purging phase; NumtS within contigs with an occupancy percentage greater than 80 were discarded.

**Common name**	**Species release**	**Total calls**	**# Passed**	**% Passed**
C.elegans	ce6	1	1	100.00
C.briggsae	cb3	59	1	1.69
Pristionchus	priPac1	52	52	100.00
Ciona	ci2	46	46	100.00
Drosophila	dm3	43	43	100.00
Tetraodon	tetNig2	5	5	100.00
Fugu	fr2	7	7	100.00
Chicken	galGal3	21	21	100.00
Platypus	ornAna1	4431	1915	43.22
Opossum	monDom5	939	939	100.00
Elephant	loxAfr3	200	198	99.00
Dog	canFam2	302	299	99.01
Horse	equCab2	278	277	99.64
Cow	bosTau6	432	432	100.00
Pig	susScr2	403	403	100.00
Rabbit	oryCun2	242	119	49.17
Rat	rn4	81	78	96.30
Mouse	mm9	169	169	100.00
Macaque	rheMac2	745	745	100.00
Orangutan	ponAbe2	902	734	81.37
Gorilla	gogGor3	675	674	99.85
Chimpanzee	panTro3	917	809	88.22
Human	hg19	764	755	98.82

Supplementary Table S2. Platypus NumtS spanning the entire contig lengths.

Query id	Subject id Contig #	Length	% similarity	alignment length	Mis matches	gap	q. start	q. end	s. start	s. end	e-value	bit score
Platypus mtDNA	105885	850	96.15	857	24	7	3240	4094	1	850	0	1373
"	107882	892	97.76	892	6	10	132	1009	1	892	0	1487
"	115640	875	97.72	876	5	11	1121	1982	1	875	0	1454
"	121664	826	99.03	827	1	6	274	1094	1	826	0	1434
"	128337	905	93.92	905	18	18	7149	8016	1	905	0	1337
"	136199	846	95.61	865	18	11	2746	3609	1	846	0	1357
"	139677	1101	92.61	1109	56	15	1880	2970	1	1101	0	1586
"	148405	785	96.43	785	2	5	517	1275	1	785	0	1290
"	148850	830	96.92	843	13	9	2790	3632	1	830	0	1375
"	153271	878	98.52	878	7	5	5669	6540	1	878	0	1508
"	154546	880	96.7	880	11	11	5541	6402	1	880	0	1424
"	155396	855	96.61	855	16	11	4242	5083	1	855	0	1373
"	156589	846	97.28	847	9	11	1535	2368	1	846	0	1388
"	163489	888	98.31	888	9	5	5775	6656	1	888	0	1517
"	168119	892	96.3	892	11	15	12889	13758	1	892	0	1413
"	175199	835	97.37	835	7	9	6542	7361	1	835	0	1380
"	177811	618	91.94	620	43	7	404	1018	1	618	0	868
"	180295	573	93.37	573	22	7	6871	7427	1	573	0	845
"	181190	448	94.65	449	21	2	10562	11008	1	448	0	695
"	191827	867	97.36	870	10	10	2457	3316	1	867	0	1433
"	193349	844	96.98	861	9	13	2388	3248	1	844	0	1393
"	194434	838	97.73	838	5	12	5225	6048	1	838	0	1384
"	195525	779	96.72	793	12	8	2548	3340	1	779	0	1290
"	196112	825	98.19	827	4	8	203	1020	1	825	0	1398
"	197949	861	97.33	863	4	15	901	1746	1	861	0	1402
"	200662	809	97.56	819	10	5	12441	13259	1	809	0	1348
"	204050	813	97.17	813	6	14	12218	13013	1	813	0	1290
"	204559	825	97.7	827	8	7	7526	8343	1	825	0	1384
"	204569	829	96.39	832	11	15	2070	2885	1	829	0	1315
"	206693	890	95.51	891	11	18	5059	5921	1	890	0	1371
"	207231	857	96.97	857	7	17	9248	10085	1	857	0	1370
"	207613	702	89.05	703	62	10	6115	6803	1	702	0	890

"	209369	866	97.69	867	11	6	11676	12534	1	866	0	1454
"	209376	862	97.1	862	16	6	13288	14140	1	862	0	1398
"	209555	667	91.02	668	35	9	8621	9264	1	667	0	917
"	209946	570	94.27	576	24	5	14032	14604	1	570	0	875
"	210303	817	98.66	818	4	5	9424	10235	1	817	0	1409
"	211368	825	97.09	826	12	8	8690	9504	1	825	0	1357
"	211589	487	89.53	487	39	8	5871	6345	1	487	7E-175	623
"	212715	570	86.83	577	55	10	8616	9178	1	570	0	672
"	213702	777	94.08	777	41	5	9316	10087	1	777	0	1177
"	214860	892	93.39	892	25	25	3183	4040	1	892	0	1261
"	215336	792	92.18	793	22	12	6424	7177	1	792	0	1133
"	216386	800	93.38	800	40	12	5118	5904	1	800	0	1162
"	216586	865	98.27	865	8	5	5478	6335	1	865	0	1476
"	216751	829	95.78	829	15	16	7116	7924	1	829	0	1283
"	217250	865	96.07	865	19	7	6866	7715	1	865	0	1389
"	219381	843	97.27	844	8	13	6708	7537	1	843	0	1373
"	220095	822	97.45	825	13	6	10309	11128	1	822	0	1373
"	221786	844	95.97	844	16	14	8579	9404	1	844	0	1323
"	222593	842	95.97	843	19	9	3168	3996	1	842	0	1341
"	222843	838	97.98	841	5	7	1149	1980	1	838	0	1420
"	223169	817	96.89	835	8	13	8154	8988	1	817	0	1346
"	225533	822	94.89	822	21	17	10054	10854	1	822	0	1211
"	225576	782	97.11	795	7	13	5272	6063	1	782	0	1287
"	228088	562	88.99	563	38	10	11619	12158	1	562	0	713
"	230511	863	98.38	866	4	9	13826	14684	1	863	0	1442
"	232177	356	98.03	356	4	1	9580	9932	1	356	2E-170	609
"	232690	752	90.09	757	64	4	14213	14963	1	752	0	1020
"	234347	828	96.38	828	12	12	13189	13998	1	828	0	1296
"	236849	660	84.82	685	35	30	9898	10538	1	660	0	672
"	238211	781	91.56	782	56	7	8155	8927	1	781	0	1090
"	239448	819	97.11	831	12	8	11232	12062	1	819	0	1366
"	241842	808	97.68	819	7	8	857	1674	1	808	0	1366
"	242887	936	90.1	939	85	6	5480	6413	1	936	0	1254
"	244188	848	96.97	859	15	8	10819	11677	1	848	0	1406
"	244909	881	96.94	882	7	14	1508	2370	1	881	0	1424
"	245137	847	95.4	847	17	13	8691	9515	1	847	0	1314
"	247361	810	90.14	811	71	9	11173	11975	1	810	0	1070

"	248759	612	92.65	612	30	8	447	1043	1	612	0	879
"	250303	573	87.54	578	55	9	9828	10393	1	573	0	690
"	252306	578	90.48	578	44	6	10804	11370	1	578	0	778
"	255355	500	93.6	500	29	2	8747	9243	1	500	0	751
"	255368	572	93.18	572	31	6	14309	14872	1	572	0	836
"	255458	818	97.56	819	7	12	1163	1969	1	818	0	1344
"	255628	386	92.78	388	23	3	8666	9050	1	386	2E-157	565
"	255630	685	94.03	687	28	7	6342	7017	1	685	0	1034
"	255631	831	96.52	833	11	13	2625	3441	1	831	0	1330
"	255840	526	94.11	526	18	3	922	1434	1	526	0	807
"	257085	823	97.69	823	12	4	11306	12121	1	823	0	1388
"	257183	667	93.7	667	32	7	12434	13090	1	667	0	993
"	259383	484	93.85	488	17	5	14927	15405	1	484	0	735
"	261355	728	93.69	729	32	12	1752	2467	1	728	0	1067
"	262340	844	96.34	847	20	7	2753	3591	1	844	0	1366
"	263194	887	97.41	887	22	1	151	1036	1	887	0	1492
"	264873	623	91.72	628	34	7	9106	9720	1	623	0	883
"	264930	616	86.41	618	39	20	5122	5696	1	616	0	686
"	265109	566	92.76	566	40	1	221	785	1	566	0	832
"	266032	526	86.73	535	44	12	8806	9322	1	526	1E-172	616
"	266034	719	91.38	719	27	10	5066	5749	1	719	0	1003
"	266392	632	94.78	632	10	6	9143	9751	1	632	0	985
"	269844	789	96.45	789	14	10	11267	12041	1	789	0	1265
"	270181	867	98.16	868	9	6	9469	10330	1	867	0	1447
"	270329	794	98.25	802	6	7	2298	3099	1	794	0	1359
"	270880	635	91.35	636	49	4	6092	6722	1	635	0	886
"	273384	446	88.2	449	42	6	3060	3500	1	446	3E-154	554
"	274004	812	97.06	815	8	14	2366	3167	1	812	0	1314
"	275491	859	98.17	872	3	9	701	1572	1	859	0	1472
"	276875	692	85.09	704	60	20	10057	10727	1	692	0	722
"	277322	463	94.4	464	23	3	5428	5889	1	463	0	710
"	277872	827	97.24	834	14	7	12070	12901	1	827	0	1352
"	277944	807	97.21	824	6	13	1223	2046	1	807	0	1339
"	96330	903	95.57	903	22	13	3491	4375	1	903	0	1406

Supplementary Table S3. BlastN high scoring pair number matrix. The number of High Scoring Pairs (HSPs) resulted from "crossed" BlastN with basic statistics. In each filed the first value is the number of purged HSPs, while the second, is the raw number of HSPs. The principal diagonal reports the number of intraspecies HSPs (the same value is reported in the last column of Table1).

**Nuclear genome**	**chrM Pristionchus**	**chrM C.briggsae**	**chrM C.elegans**	**chrM Drosophila**	**chrM Ciona**	**chrM Tetraodon**	**chrM Fugu**	**chrM Chicken**	**chrM Platypus**	**chrM Opossum**	**chrM Elephant**	**chrM Dog**	**chrM Horse**	**chrM Cow**	**chrM Pig**	**chrM Rabbit**	**chrM Rat**	**chrM Mouse**	**chrM Macaque**	**chrM Orangutan**	**chrM Gorilla**	**chrM Chimpanzee**	**chrM Human**
**pristionchus**	**0**<br>**52**	0<br>28	0<br>30	0<br>1	0<br>6	0<br>0	0<br>0	0<br>0	0<br>0	0<br>0	0<br>2	0<br>1	0<br>0	0<br>0	0<br>0	0<br>1	0<br>0	0<br>0	0<br>0	0<br>0	0<br>0	0<br>0	0<br>0
**C. briggsae**	2<br>42	**1**<br>**59**	2<br>48	0<br>10	0<br>11	0<br>2	0<br>0	0<br>0	0<br>2	0<br>4	0<br>2	0<br>2	0<br>2	0<br>4	0<br>2	0<br>7	0<br>2	0<br>2	0<br>4	0<br>2	0<br>4	0<br>4	0<br>2
**C. elegans**	1<br>1	1<br>1	**1**<br>**1**	0<br>0	0<br>0	0<br>0	0<br>0	0<br>0	0<br>0	0<br>0	0<br>0	0<br>0	0<br>0	0<br>0	0<br>0	0<br>0	0<br>0	0<br>0	0<br>0	0<br>0	0<br>0	0<br>0	0<br>0
**Drosophila**	9<br>9	9<br>9	9<br>9	**43**<br>**43**	11<br>11	4<br>4	7<br>7	8<br>8	10<br>10	10<br>10	8<br>8	8<br>8	8<br>8	12<br>12	8<br>8	7<br>7	9<br>9	11<br>11	8<br>8	7<br>7	11<br>11	9<br>9	8<br>8
**Ciona**	6<br>6	4<br>4	4<br>4	9<br>9	**46**<br>**46**	2<br>2	2<br>2	0<br>0	4<br>4	5<br>5	5<br>5	3<br>3	1<br>1	1<br>1	2<br>2	1<br>1	2<br>2	4<br>4	1<br>1	1<br>1	0<br>0	1<br>1	2<br>2
**Tetraodon**	0<br>0	0<br>0	0<br>0	1<br>3	0<br>0	**0**<br>**5**	0<br>5	0<br>5	0<br>6	0<br>3	0<br>4	0<br>6	0<br>4	0<br>4	0<br>4	0<br>4	0<br>4	0<br>4	1<br>4	0<br>6	0<br>4	0<br>5	0<br>4
**Fugu**	0<br>2	0<br>0	0<br>0	0<br>9	0<br>1	0<br>6	**0**<br>**7**	0<br>8	0<br>7	0<br>7	0<br>7	0<br>10	0<br>8	0<br>7	0<br>7	0<br>6	0<br>8	0<br>7	0<br>7	0<br>10	0<br>9	0<br>8	0<br>7
**Chicken**	0<br>0	0<br>0	0<br>0	0<br>0	0<br>0	5<br>5	6<br>6	**21**<br>**21**	2<br>2	3<br>3	6<br>6	5<br>5	3<br>3	7<br>7	4<br>4	6<br>6	6<br>6	2<br>2	4<br>4	4<br>4	5<br>5	2<br>2	3<br>3
**Platypus**	391<br>447	419<br>419	476<br>544	1532<br>1534	427<br>427	1664<br>2897	1369<br>2733	1416<br>2593	**1915**<br>**4412**	1578<br>3269	1309<br>3023	1135<br>3002	1089<br>2999	1027<br>3049	1089<br>3124	1037<br>3028	1524<br>3236	1205<br>2993	1283<br>2864	1457<br>2929	1355<br>3011	1464<br>3043	1424<br>2983
**Opossum**	83<br>83	53<br>53	75<br>75	332<br>332	115<br>115	542<br>542	539<br>539	534<br>534	679<br>679	**939**<br>**939**	613<br>1075	635<br>635	638<br>638	663<br>663	660<br>660	659<br>659	685<br>685	689<br>689	593<br>593	585<br>585	604<br>1144	621<br>621	569<br>569
**Elephant**	35<br>35	12<br>12	30<br>30	150<br>150	46<br>46	184<br>184	164<br>164	177<br>177	185<br>185	179<br>179	**198**<br>**198**	197<br>197	204<br>204	217<br>217	207<br>207	192<br>192	194<br>194	196<br>196	171<br>171	209<br>209	185<br>185	199<br>199	176<br>176
**Dog**	14<br>14	2<br>2	7<br>7	53<br>53	12<br>12	108<br>108	106<br>107	99<br>100	152<br>152	153<br>154	169<br>171	**229**<br>**302**	244<br>246	226<br>228	251<br>253	207<br>208	197<br>198	187<br>188	152<br>153	158<br>160	158<br>160	171<br>173	165<br>167
**Horse**	5<br>5	4<br>4	2<br>2	24<br>24	9<br>9	79<br>79	84<br>84	70<br>70	99<br>99	122<br>122	126<br>126	213<br>213	**277**<br>**278**	211<br>211	219<br>219	172<br>172	158<br>158	168<br>168	131<br>131	134<br>134	131<br>131	131<br>131	130<br>130
**Cow**	23<br>23	10<br>10	20<br>20	72<br>72	20<br>20	181<br>181	171<br>171	161<br>161	257<br>258	275<br>275	301<br>301	389<br>389	428<br>428	**432**<br>**432**	433<br>434	336<br>337	330<br>331	339<br>339	264<br>264	265<br>265	275<br>275	272<br>272	273<br>273
**Pig**	7<br>7	4<br>4	1<br>1	58<br>58	9<br>9	145<br>145	136<br>136	122<br>122	205<br>205	227<br>227	248<br>248	342<br>342	381<br>381	367<br>367	**403**<br>**403**	295<br>295	269<br>269	274<br>274	216<br>216	221<br>221	242<br>242	238<br>238	232<br>232
**Rabbit**	2<br>16	0<br>6	0<br>9	10<br>48	1<br>12	31<br>95	32<br>91	32<br>93	61<br>121	67<br>152	77<br>161	95<br>172	106<br>207	107<br>188	99<br>192	**119**<br>**239**	78<br>172	83<br>175	55<br>112	66<br>141	62<br>136	64<br>134	63<br>131

**Rat**	1<br>1	0<br>0	0<br>0	3<br>4	2<br>2	14<br>15	17<br>18	12<br>12	31<br>32	33<br>34	31<br>34	58<br>60	48<br>50	59<br>62	50<br>52	44<br>46	**78**<br>**81**	86<br>89	25<br>27	29<br>30	34<br>38	32<br>34	33<br>35
**Mouse**	8<br>8	7<br>7	6<br>6	31<br>31	10<br>10	44<br>44	46<br>46	47<br>47	71<br>71	87<br>87	80<br>80	107<br>107	112<br>112	107<br>107	116<br>116	114<br>114	153<br>153	**169**<br>**169**	85<br>85	77<br>77	90<br>90	87<br>87	87<br>87
**Macaque**	52<br>52	21<br>21	31<br>31	197<br>197	53<br>53	493<br>493	503<br>503	487<br>487	561<br>561	594<br>594	620<br>620	705<br>705	755<br>755	712<br>712	745<br>745	710<br>710	705<br>705	695<br>695	**745**<br>**745**	778<br>778	763<br>763	776<br>776	737<br>737
**Orangutan**	17<br>38	6<br>14	12<br>23	123<br>182	39<br>62	396<br>508	406<br>522	401<br>515	488<br>597	542<br>665	558<br>696	628<br>760	674<br>810	660<br>801	692<br>835	653<br>792	615<br>745	602<br>735	678<br>840	**734**<br>**902**	716<br>872	732<br>892	717<br>871
**Gorilla**	20<br>20	5<br>5	15<br>15	145<br>145	34<br>34	397<br>397	414<br>414	396<br>396	467<br>467	519<br>519	525<br>525	598<br>598	650<br>650	642<br>642	660<br>660	617<br>617	596<br>596	600<br>600	685<br>685	706<br>706	**674**<br>**674**	705<br>705	681<br>681
**Chimpanzee**	37<br>42	9<br>10	25<br>39	184<br>236	46<br>69	503<br>587	515<br>591	501<br>580	553<br>640	583<br>676	596<br>687	666<br>761	725<br>807	692<br>781	743<br>839	703<br>796	665<br>742	687<br>779	730<br>824	799<br>915	767<br>861	**809**<br>**914**	773<br>874
**Human**	45<br>45	10<br>10	27<br>29	201<br>208	74<br>78	478<br>488	495<br>502	481<br>488	548<br>556	608<br>616	574<br>583	680<br>690	737<br>744	699<br>708	737<br>746	701<br>710	661<br>668	658<br>666	723<br>731	795<br>806	769<br>777	771<br>779	**755**<br>**764**

Supplementary Table S4. BlastN High Scoring Pairs HSP length and similarity matrices after purging. A HSP mean length resulted from “crossed” BlastN with basic statistics, after the purging phase. B HSPs mean similarities resulted from “crossed” BlastN with basic statistics, after the purging phase.

**Nuclear genomes**	**chrM *C.elegans***	**chrM *C.briggsae***	**chrM *Pristionchus***	**chrM *Ciona***	**chrM *Drosophila***	**chrM *Tetraodon***	**chrM *Fugu***	**chrM *Chicken***	**chrM *Platypus***	**chrM *Opossum***	***chrM elephant***	**chrM *dog***	**chrM *horse***	**chrM *Cow***	**chrM *Pig***	**chrM *Rabbit***	**chrM *Rat***	**chrM *mouse***	**chrM *Macaque***	**chrM *Orangutan***	***chrM Gorilla***	**chrM *Chimpanzee***	**chrM *Human***	**Average**	**Standard deviation**
**A - NumtS Length**																									
**C.elegans**	126	123	123	0	0	0	0	0	0	0	0	0	0	0	0	0	0	0	0	0	0	0	0	16	43
**C.briggsae**	89	247	74	0	0	0	0	0	0	0	0	0	535	303	0	0	0	0	0	0	0	0	0	54	132
**Pristionchus**	0	0	0	0	0	0	0	0	0	0	0	0	0	0	0	0	0	0	0	0	0	0	0	0	0
**Ciona**	588	308	281	261	279	371	387	0	450	464	326	483	1240	1196	635	1160	654	426	1208	713	0	627	431	543	357
**Drosophila**	289	215	246	219	248	604	501	441	498	460	562	452	493	370	503	546	444	447	451	433	344	336	421	414	112
**Tetraodon**	0	0	0	0	71	0	0	0	0	0	0	0	0	0	0	0	0	0	40	0	0	0	0	5	17
**Fugu**	0	0	0	0	0	0	0	0	0	0	0	0	0	0	0	0	0	0	0	0	0	0	0	0	0
**Chicken**	0	0	0	0	0	295	326	287	156	138	165	152	171	199	275	231	150	129	171	203	195	132	128	152	99
**Platypus**	247	181	248	245	260	379	403	401	228	392	424	438	440	429	417	436	393	437	450	385	407	384	423	367	84
**Opossum**	311	140	253	180	248	581	630	578	727	681	732	717	744	731	742	728	692	691	692	659	701	668	712	589	202
**Elephant**	411	152	423	194	277	1061	1231	1043	1240	1273	1383	1289	1275	1194	1266	1334	1300	1255	1420	1130	1310	1211	1409	1047	421
**Dog**	354	152	269	253	278	481	495	470	536	519	538	412	481	498	459	500	479	503	540	516	553	490	538	448	110
**Horse**	347	68	311	195	277	575	560	598	676	569	605	439	369	433	439	505	496	478	580	531	576	572	578	468	149
**Cow**	143	66	183	188	261	499	553	548	610	563	610	584	579	571	561	593	548	546	630	588	615	611	615	490	178
**Pig**	1014	223	198	254	280	495	529	548	564	532	556	508	500	501	463	524	519	511	557	515	535	515	544	495	160
**Rabbit**	0	95	78	309	258	410	411	367	357	384	400	397	371	364	379	346	396	398	419	378	413	389	388	335	116
**Rat**	0	0	50	114	191	216	230	177	238	218	225	196	243	221	240	241	280	268	207	192	227	175	188	189	77
**Mouse**	287	150	220	248	265	471	513	456	480	408	469	401	412	406	391	411	391	359	399	439	408	425	435	384	91
**Macaque**	233	99	190	241	256	615	624	628	774	711	784	724	707	736	706	742	680	694	733	661	711	692	740	595	217
**Orangutan**	243	53	201	172	254	475	494	485	618	549	612	578	565	564	540	569	549	556	552	498	536	522	539	466	159
**Gorilla**	235	63	195	172	243	477	475	489	624	551	641	588	561	562	548	580	543	543	536	500	541	525	545	467	162
**Chimpanzee**	349	63	263	205	255	562	625	608	834	774	862	800	758	794	739	778	761	731	768	671	726	687	734	624	229
**Human**	289	59	240	189	275	652	657	675	843	724	903	785	739	788	739	780	758	756	764	667	721	714	746	629	236

B - NumtS Similarity																									
**C.elegans**	96	86	80	0	0	0	0	0	0	0	0	0	0	0	0	0	0	0	0	0	0	0	0	87	8
**C.briggsae**	81	100	82	0	0	0	0	0	0	0	0	0	0	0	0	0	0	0	0	0	0	0	0	88	10
**Pristionchus**	0	0	0	0	0	0	0	0	0	0	0	0	0	0	0	0	0	0	0	0	0	0	0	0	0
**Ciona**	69	69	73	98	74	69	71	0	72	71	73	75	64	67	73	66	72	72	64	64	0	67	71	71	7
**Drosophila**	73	72	75	76	98	72	69	75	71	73	71	75	72	73	72	72	74	77	71	72	74	73	72	74	6
**Fugu**	0	0	0	0	0	0	0	0	0	0	0	0	0	0	0	0	0	0	0	0	0	0	0	0	0
**Tetraodon**	0	0	0	0	77	0	0	0	0	0	0	0	0	0	0	0	0	0	90	0	0	0	0	84	9
**Chicken**	0	0	0	0	0	73	70	78	71	74	73	73	71	71	70	71	75	74	70	71	70	75	74	72	2
**Platypus**	76	74	75	73	74	72	73	73	92	75	73	75	75	75	74	74	76	75	72	73	73	73	73	75	4
**Opossum**	74	77	74	75	74	71	71	71	73	80	72	73	73	73	73	73	74	74	71	71	71	72	71	73	2
**Elephant**	76	74	69	75	74	71	71	71	72	73	83	75	75	76	75	75	74	74	73	73	73	73	72	74	3
**Dog**	70	72	75	72	73	73	73	73	74	75	75	83	79	78	78	77	77	76	75	75	74	75	75	75	3
**Horse**	69	80	74	74	73	73	74	73	74	75	76	79	83	79	79	77	77	77	75	76	75	76	76	76	3
**Cow**	78	81	75	73	73	71	72	71	72	73	73	74	74	76	75	73	73	73	72	72	72	73	73	74	2
**Pig**	66	70	78	71	71	73	73	73	73	73	74	76	76	77	80	75	75	75	73	74	74	74	74	74	3
**Rabbit**	0	77	80	70	73	73	72	73	75	73	73	75	75	75	74	76	75	74	73	74	74	74	75	74	2
**Rat**	0	0	84	73	73	73	74	77	73	76	75	77	75	75	75	75	79	78	75	75	75	75	76	76	2
**Mouse**	77	78	78	71	74	72	73	74	74	75	74	75	75	75	75	75	78	81	75	74	74	74	74	75	2
**Macaque**	73	80	75	71	73	72	72	72	72	73	73	75	75	75	75	75	75	74	79	77	77	78	77	75	2
**Orangutan**	74	87	75	73	73	72	72	72	73	74	73	75	76	76	76	76	75	75	77	79	78	79	79	76	3
**Gorilla**	72	85	74	73	73	73	73	72	73	74	73	75	76	76	76	76	75	75	77	78	79	79	78	75	3
**Chimpanzee**	70	84	73	72	73	72	72	71	73	73	73	75	76	76	76	76	75	74	77	78	79	80	79	75	3
**Human**	72	85	73	73	73	72	72	72	74	74	73	75	77	76	76	76	75	75	77	78	79	79	79	75	3

Supplementary Table S5. Count of repetitive elements found in NumtS loci or 5’ or 3’ flanking regions. Number of NumtS containing repetitive elements either in NumtS loci or in flanking regions (2 kbp apart), for each analysed species.

Species	Release	# NumtS	# NumtS loci with repmask elements	# NumtS 5' flank with repmask elements	# NumtS 3' flank with repmask elements
C.elegans	priPac1	52	0	0	0
C.briggsae	cb3	59	2	0	0
Pristionchus	ce6	1	0	0	0
Ciona	dm3	44	2	0	1
Drosophila	ci2	45	2	0	0
Fugu	tetNig2	4	0	0	0
Tetraodon	fr2	6	0	0	0
Chicken	galGal3	21	0	0	2
Platypus	ornAna1	1914	30	43	4
Opossum	monDom5	938	13	138	88
Elephant	loxAfr3	197	6	17	19
Dog	canFam2	329	4	20	16
Horse	equCab2	291	2	23	12
Cow	bosTau6	431	4	22	26
Pig	susScr2	416	1	18	19
Rabbit	oryCun2	238	0	6	10
Rat	rn4	77	0	6	9
Mouse	mm9	172	6	20	18
Macaque	rheMac2	751	1	61	48
Orangutan	ponAbe2	733	30	79	68
Gorilla	gorGor3	673	21	65	67
Chimpanzee	panTro3	872	153	71	71
Human	hg19	766	19	84	86

Supplementary Table S6. UCSC tracks links. Link list for all UCSC NumtS tracks.
NB: to be run activating in the browser options a newly incognito window

##Chimpanzee_tracks	http://genome.ucsc.edu/cgi-bin/hgTracks?db=panTro3&hgt.reset=1&position=chr1%3A5522552-5522623&hgt.customText=http://webservice.cloud.ba.infn.it/public/ucsc_tracks/chimp/all_chimp_tracks.txt
##Rabbit_tracks	http://genome.ucsc.edu/cgi-bin/hgTracks?db=oryCun2&hgt.reset=1&position=chr1%3A42316325-42316405&hgt.customText=http://webservice.cloud.ba.infn.it/public/ucsc_tracks/rabbit/all_rabbit_tracks.txt
##Elephant_tracks	http://genome.ucsc.edu/cgi-bin/hgTracks?db=loxAfr3&hgt.reset=1&position=scaffold_0%3A92246551-92247107&hgt.customText=http://webservice.cloud.ba.infn.it/public/ucsc_tracks/elephant/all_elephant_tracks.txt
##Gorilla_trakcs	http://genome.ucsc.edu/cgi-bin/hgTracks?db=gorGor3&hgt.reset=1&position=CABD02429275%3A2576-2729&hgt.customText=http://webservice.cloud.ba.infn.it/public/ucsc_tracks/gorilla/all_gorilla_tracks.txt
##Horse_tracks	http://genome.ucsc.edu/cgi-bin/hgTracks?db=equCab2&hgt.reset=1&position=chr1%3A21812131-21813046&hgt.customText=http://webservice.cloud.ba.infn.it/public/ucsc_tracks/horse/all_horse_tracks.txt
##Mouse_tracks	http://genome.ucsc.edu/cgi-bin/hgTracks?db=mm9&hgt.reset=1&position=chr1%3A20936147-20936971&hgt.customText=http://webservice.cloud.ba.infn.it/public/ucsc_tracks/mouse/all_mouse_tracks.txt

##Macaque_tracks	http://genome.ucsc.edu/cgi-bin/hgTracks?db=rheMac2&hgt.reset=1&position=chr1%3A5484600-5484695&hgt.customText=http://webservice.cloud.ba.infn.it/public/ucsc_tracks/macaca/all_macaca_tracks.txt
##Platypus_tracks	http://genome.ucsc.edu/cgi-bin/hgTracks?db=ornAna1&hgt.reset=1&position=chr1%3A386695-386775&hgt.customText=http://webservice.cloud.ba.infn.it/public/ucsc_tracks/platypus/all_platypus_tracks.txt
##Orangutan_tracks	http://genome.ucsc.edu/cgi-bin/hgTracks?db=ponAbe2&hgt.reset=1&position=chr1%3A68516-68624&hgt.customText=http://webservice.cloud.ba.infn.it/public/ucsc_tracks/orangutan/all_orangutan_tracks.txt
##Rat_tracks	http://genome.ucsc.edu/cgi-bin/hgTracks?db=rn4&hgt.reset=1&position=chr1%3A101684758-101684916&hgt.customText=http://webservice.cloud.ba.infn.it/public/ucsc_tracks/rattus/all_rattus_tracks.txt
##Drosophyla_tracks	http://genome.ucsc.edu/cgi-bin/hgTracks?db=dm3&hgt.reset=1&position=chr2L%3A21794052-21794161&hgt.customText=http://webservice.cloud.ba.infn.it/public/ucsc_tracks/drosophyla/all_drosophyla_tracks.txt
##Dog_tracks	http://genome.ucsc.edu/cgi-bin/hgTracks?db=canFam2&hgt.reset=1&position=chr1%3A75586562-75586634&hgt.customText=http://webservice.cloud.ba.infn.it/public/ucsc_tracks/dog/all_dog_tracks.txt

##Pig_tracks	http://genome.ucsc.edu/cgi-bin/hgTracks?db=susScr2&hgt.reset=1&position=chr1%3A43384304-43384343&hgt.customText=http://webservice.cloud.ba.infn.it/public/ucsc_tracks/pig/all_pig_tracks.txt
##Chicken_tracks	http://genome.ucsc.edu/cgi-bin/hgTracks?db=galGal3&hgt.reset=1&position=chr1%3A11510176-11510610&hgt.customText=http://webservice.cloud.ba.infn.it/public/ucsc_tracks/chicken/all_chicken_tracks.txt
##Opossum_tracks	http://genome.ucsc.edu/cgi-bin/hgTracks?db=monDom5&hgt.reset=1&position=chr1%3A9991652-9992107&hgt.customText=http://webservice.cloud.ba.infn.it/public/ucsc_tracks/opossum/all_opossum_tracks.txt

Supplementary Table S7. Hmmer NumtS dating. The coefficient allowing the discrimination between fragments more similar to mitochondrial sequences and those more similar to nuclear ones was calculated as the difference between the score on mitochondrion profile and the score on flanking region profiles. The HMM mitochondrial and flanking score differences are meaningful of quite new insertion events, if positive or old if negative.

NumtS name	chr	Nuclear start	Nuclear end	score vs. flanks	score vs mt.	score diff.
Oan_NumtS_3958	Ultra334	83840	83885	30.3	-5.4	-35.7
Oan_NumtS_3234	Contig25573	16519	17120	5.5	-7.9	-13.4
Oan_NumtS_3661	Contig52602	1061	1330	1.3	-5.9	-7.2
Oan_NumtS_3024	Contig23952	3253	3582	-0.1	-7	-6.9
Oan_NumtS_3576	Contig28021	2598	2755	0.3	-6	-6.3
Oan_NumtS_3965	Ultra341	5317918	5317995	-0.9	-7.2	-6.3
Oan_NumtS_3629	Contig393	246200	246317	0.9	-4.8	-5.7
Oan_NumtS_3385	Contig26960	2587	2651	-1	-6.4	-5.4
Oan_NumtS_4003	Ultra483	133594	133987	5.3	0	-5.3
Oan_NumtS_0267	Contig11450	9319	9420	-1.5	-6.7	-5.2
Oan_NumtS_3256	Contig25790	4590	4673	-1.4	-6.5	-5.1
Oan_NumtS_4006	Ultra483	4593672	4593738	-1.6	-6.7	-5.1
Oan_NumtS_4002	Ultra483	132717	133561	3.5	-1.4	-4.9
Oan_NumtS_4011	Ultra51	360171	360278	0.1	-4.6	-4.7
Oan_NumtS_0005	chr1	24124250	24124329	-1.8	-6.4	-4.6
Oan_NumtS_0963	Contig15437	10297	10369	-2	-6.6	-4.6
Oan_NumtS_3597	Contig2959	13649	13727	-2.4	-6.5	-4.1
Oan_NumtS_0032	chr3	54786592	54786727	-0.4	-4.2	-3.8
Oan_NumtS_0825	Contig14973	11331	11431	-2.6	-5.1	-2.5
Oan_NumtS_0007	chr1	32610541	32610726	-0.6	-2.8	-2.2
Oan_NumtS_3959	Ultra334	83958	84064	-2.1	-3.1	-1
Oan_NumtS_3790	Contig8670	5938	6394	-3.1	-3.4	-0.3
Oan_NumtS_3224	Contig255384	1004	1090	63.7	70.9	7.2
Oan_NumtS_3645	Contig45811	1792	1848	-1.9	14.7	16.6
Oan_NumtS_3946	Ultra292	895124	895168	-4.3	16.7	21
Oan_NumtS_0011	chr10	2585389	2585426	-3.1	21	24.1
Oan_NumtS_0085	chrX5	23020990	23021051	-3.2	25.7	28.9
Oan_NumtS_3964	Ultra341	2638918	2638979	-2.1	27.5	29.6
Oan_NumtS_3814	Contig91506	1206	1270	0.3	31.7	31.4
Oan_NumtS_0629	Contig13773	7806	7866	-3.1	35.5	38.6
Oan_NumtS_0038	chr4	17188496	17188597	-3	42.5	45.5
Oan_NumtS_3459	Contig2740	20182	20296	-2.9	44.3	47.2
Oan_NumtS_0037	chr4	12862525	12862667	-2.8	70.4	73.2
Oan_NumtS_3666	Contig57515	1490	1610	-1.6	87.3	88.9
Oan_NumtS_3690	Contig67895	3502	3805	-3	132.3	135.3
Oan_NumtS_3637	Contig40385	2028	2366	0.7	152.1	151.4
Oan_NumtS_0130	Contig10522	33296	33514	-0.8	178	178.8

Oan_NumtS_0084	chrX5	9939063	9939266	-0.2	200.1	200.3
Oan_NumtS_3669	Contig58952	2255	2444	-0.4	207.9	208.3
Oan_NumtS_0129	Contig10522	31799	32045	-0.4	217.1	217.5
Oan_NumtS_4018	Ultra514	4101189	4101412	0.8	260.2	259.4
Oan_NumtS_3624	Contig37374	1010	1446	3.4	431.2	427.8
Oan_NumtS_0283	Contig116526	2275	3053	3.4	454.1	450.7
